# Emergency Severity Index Stratified by Post-Triage Lactate: Association with In-Hospital Mortality in Admitted Patients

**DOI:** 10.5811/westjem.62115

**Published:** 2026-08-23

**Authors:** Gregory Mansella, Valentin Klotzbücher, Roland Bingisser, Christian H. Nickel

**Affiliations:** * University Hospital Basel, Department of Emergency Medicine, Basel, Switzerland; † University of Basel and University Hospital Basel, Department of Clinical Research, Basel, Switzerland

## Abstract

**Introduction:**

The Emergency Severity Index (ESI) is a widely used triage tool in emergency departments (EDs) and is associated with adverse outcomes. Lactate has also been associated with mortality in various ED populations. This study evaluated whether lactate levels measured after triage at the treating physician’s discretion are associated with in-hospital mortality across ESI levels in admitted ED patients.

**Methods:**

This study is a secondary analysis of prospectively collected data from adult patients presenting to the ED of a Swiss tertiary care center in 2013, 2015, 2017, and 2019. The study included admitted patients who underwent post-triage lactate measurement at the treating physician’s discretion. Only the first lactate value measured in the ED was analyzed and categorized as < 2.0, 2.0–3.9, and ≥ 4.0 mmol/L. The primary outcome was in-hospital mortality; secondary outcomes were 30-day mortality and intensive care unit (ICU) admission. Associations were assessed using multivariable logistic regression models adjusted for age and sex. Model discrimination was compared using receiver operating characteristic curve analysis.

**Results:**

Among 3,909 patients admitted from the ED who had a lactate drawn (of 17,327 ED presentations during the study period; 22.6%), the median age was 72 years and 2,062 (52.8%) were male. In-hospital mortality occurred in 168 (4.3%), 30-day mortality in 258 (6.6%), and ICU admission in 698 (17.9%). In ESI levels 2 (n = 1,826) and 3 (n = 1,722), crude in-hospital mortality was similar (59 [3.2%] and 44 [2.6%], respectively), but varied after stratification by lactate level. In ESI level 2 patients, in-hospital mortality occurred in 24 (2.0%) among those with lactate < 2.0 mmol/L (n = 1,210) and in 18 (17.8%) among those with lactate ≥ 4.0 mmol/L (n = 101), corresponding to an age- and sex-adjusted absolute risk difference of 16.0% (95% CI, 8.8–23.3). In ESI level 3, in-hospital mortality occurred in 20 (1.6%) at lactate < 2.0 mmol/L (n = 1,254) and in 8 (15.1%) at lactate ≥ 4.0 mmol/L (n = 53), corresponding to a risk difference of 12.5% (95% CI, 3.7–21.3). Discrimination for in-hospital mortality was higher when lactate was considered alongside ESI (area under the curve [AUC] 0.844 versus 0.799; ΔAUC 0.045, 95% CI, 0.021–0.069), with smaller increases for 30-day mortality and ICU admission.

**Conclusion:**

In admitted ED patients who had lactate measured after triage at physician discretion, higher lactate levels were associated with increased in-hospital mortality across ESI levels, particularly among patients triaged as ESI levels 2 and 3.

## INTRODUCTION

Triage is one of the main tasks of an emergency department (ED), in which patients are categorized based on acuity and prognosis.^1^ The Emergency Severity Index (ESI) is a well-established, reliable, and valid five-level triage tool. Patients are classified according to presenting symptoms, resource needs, and vital signs.^2^ Despite being primarily an acuity-based triage tool, the ESI is known to be associated with hospital admission and mortality.^3^–^5^ Its discriminatory performance for adverse outcomes can be enhanced by incorporating additional parameters. Adding physician disease severity ratings to any ESI level was associated with higher mortality in ED patients.^6^ Similarly, markers of vulnerability, such as impaired mobility or the Clinical Frailty Scale, were more strongly associated with 30-day mortality than the ESI among patients aged 65 years and older, highlighting their potential utility in triage decision-making.^7^,^8^ Another study found that combining ESI with the quick Sequential Organ Failure Assessment (qSOFA) score improved discriminatory accuracy for in-hospital mortality and intensive care unit (ICU) admission compared to ESI alone.^9^ Likewise, adding the Peripheral Perfusion Index or the Shock Index to the ESI improved discrimination of high-acuity unselected ED patients.^10^

Lactate has been shown to be associated with adverse outcomes in both unselected ED patients and those presenting with specific acute conditions, including infection, sepsis, trauma, pneumonia, pulmonary embolism, and stroke.^11^–^18^ To date, no study has evaluated whether lactate levels are associated with outcomes across ESI levels in admitted ED patients. Given that incorporating lactate into triage would represent a substantial operational shift, we aimed to assess whether lactate levels obtained after triage at the discretion of the treating physician are associated with in-hospital mortality across ESI levels and may provide additional information beyond ESI.

## METHODS

### Study Design and Setting

This study is a secondary analysis based on prospectively collected data from the EMERGE cohort, which serves quality control purposes and benchmarking between Swiss EDs. The study was conducted in the ED of a tertiary care center in Switzerland, with an annual ED census of approximately 55,000 patients aged 16 years and older. Obstetric, pediatric, and ophthalmologic patients are treated in separate facilities on campus. All patients presenting to the ED were assessed for inclusion 24 hours a day, 7 days a week. Data were collected from 21 October to 11 November 2013 (EMERGE cohort 1), 1 February to 23 February 2015 (EMERGE cohort 2), 30 January to 19 February 2017 (EMERGE cohort 3), and 18 March to 20 May 2019 (EMERGE cohort 4). The study was approved by the local ethics committee (EKNZ-236/13). The results are reported in accordance with the STROBE guidelines.^19^

**Population Health Research Capsule**

What do we already know about this issue?

*Both the Emergency Severity Index (ESI) and lactate are associated with mortality in emergency department (ED) patients, but the association of lactate across ESI levels is unclear*.

What was the research question?

*Is post-triage lactate associated with mortality across ESI levels in admitted ED patients?*

What was the major finding of the study?

*In ESI 3 patients, mortality rose from 1.6% with lactate < 2 to 15.1% with ≥ 4 mmol/L (RD 12.5%; 95% CI, 3.7–21.3)*.

How does this improve population health?

*Lactate could help identify high-risk ESI 3 patients who may benefit from more timely evaluation or closer monitoring*.

### Selection Of Participants

All consecutive patients presenting to the ED during the study periods were screened for eligibility. Patients were excluded if they left without being seen, were directly referred to other departments, declined informed consent, or had repeated ED visits during the study periods (only the index visit was included). For the primary analysis, we included adult patients who were admitted to the hospital and had a lactate measurement obtained during the ED stay at the discretion of the treating physician. Patients discharged from the ED or with missing ESI or lactate values were excluded. The rationale for excluding patients discharged from the ED is their generally very low risk of mortality.^3^,^20^,^21^ In addition, lactate measurements are typically obtained in clinically sicker patients, who are more likely to require admission. Therefore, the analysis was restricted to admitted patients to focus on a higher risk population. For descriptive comparisons of ESI distributions and crude in-hospital mortality according to lactate measurement status, admitted patients without lactate measurements were included.

### Data Collection

Upon arrival, all patients were registered by the study team, and an electronic health record (EHR) was created. The study team consisted of trained medical students. Data on demographics (age, sex), ESI triage level, patient disposition (discharge, hospital admission, or ICU admission), in-hospital and 30-day mortality were prospectively and consecutively collected. Clinicians ordered nonprotocolized lactate measurements at their discretion during routine clinical care. Consequently, this secondary analysis used data extracted from the EHR for a select subset of patients who underwent testing.

#### Triage Process

All patients were triaged by a triage clinician using the German version of the ESI, version 4.^3^ The ESI stratifies patients into five acuity categories based on their presenting symptoms, anticipated resource requirements, and vital signs. High-acuity patients are classified as ESI levels 1–2, intermediate-acuity patients as ESI level 3, and low-acuity patients as ESI levels 4–5. Triage personnel assign an ESI level 1 to patients requiring immediate life-saving interventions (eg, airway or respiratory support, emergency medications, or hemodynamic interventions, such as fluid resuscitation or blood products). Patients in high-risk situations (such as those at high risk for deterioration, exhibiting acutely altered mental status, or experiencing severe pain or distress) are assigned an ESI level 2. The remaining patients are classified into ESI levels 3–5 based on the expected number of required resources (more than one in ESI level 3, one in ESI level 4, and none in ESI level 5). Examples for resources are laboratory tests, electrocardiograms, or radiographs. For patients triaged as ESI level 3, vital signs must also be measured. In the presence of danger-zone vital signs (heart rate greater than 100 beats/min, respiratory rate greater than 20 breaths/min, or oxygen saturation less than 92% in adult patients), reassignment to ESI level 2 should be considered.^22^ Notably, the fifth version of the ESI manual (released in 2023) integrated a complete set of vital signs for all low-acuity patients; however, its benefit remains uncertain.^2^,^23^

#### Lactate Measurement

Venous lactate was measured in the treatment area after triage at the discretion of the treating physician and was not part of a standardized protocol. In routine clinical practice, lactate measurement was typically performed shortly after triage, with earlier measurement in higher acuity patients (ESI levels 1–2) and later measurement in lower acuity patients depending on ED workflow. Accordingly, lactate measurements were obtained in a select subset of patients. Clinicians performed bedside lactate measurements using a point-of-care analyzer in the ED (Radiometer ABL90 FLEX PLUS; Radiometer Medical ApS, Brønshøj, Denmark). This analysis evaluated only the first lactate level obtained in the ED. To improve clinical interpretability, stratified lactate levels were used instead of continuous lactate levels. Based on thresholds used in previous studies, lactate levels were categorized as low (< 2.0 mmol/L), intermediate (2.0–3.9 mmol/L), and high (≥ 4.0 mmol/L).^24^–^27^

### Outcomes

The primary outcome was in-hospital mortality. Admission was defined as transfer from the ED to any hospital ward with a minimum stay of one night. Patients who died in the ED prior to an overnight stay were considered admitted and counted as in-hospital deaths. Secondary outcomes included 30-day mortality and ICU admission. Thirty-day mortality was defined as death occurring within 30 days after presentation to the ED. Follow-up was performed by reviewing the hospital information system; contacting patients, proxies, or primary care physicians; and consulting health insurance companies or civil registries. Patients lost to follow-up were considered alive. Admission to the ICU was defined as transfer to the ICU, intermediate care unit, or stroke unit during the hospital stay.

### Statistical Analysis

For categorical variables, data are presented as frequencies and percentages; continuous variables are reported as medians with interquartile ranges (IQRs). Given that lactate testing was performed selectively at physician discretion, patients without a lactate value were considered not to have undergone measurement rather than to have conventional missing data. Consequently, the primary analyses were restricted to admitted patients with available ESI and lactate data, and missing lactate values were not imputed. Descriptive analyses comparing patients with and without lactate measurements were performed without imputation. To assess whether lactate provided additional information beyond ESI within this selected cohort, logistic regression models were fitted for ESI alone and for the combined ESI-by-lactate specification; adjusted models included age and sex. Nested model comparisons were performed using likelihood-ratio tests (two-sided *P* < .05). Discrimination was assessed using the area under the receiver operating characteristic curve (AUC) with 95% confidence intervals (CIs). Changes in discrimination (ΔAUC) were reported with 95% CIs; statistical inference for nested models relied on likelihood-ratio tests, with ΔAUC estimates presented descriptively. Patients lost to follow-up at 30 days were coded as survivors in the primary analysis, with sensitivity analyses excluding them or recoding them as deaths (Supplementary Table S1). Due to small sample sizes, ESI levels 4 and 5 were combined. Adjusted analyses were considered primary and are presented in the main manuscript, whereas unadjusted analyses are provided in the Supplementary Materials (Supplementary Tables S2, S3, and S4 and Figure S1). Because this study was a secondary analysis of a prospectively collected cohort, no formal sample size calculation was performed a priori. The sample size was determined by the number of eligible patients available during the study periods. We did not perform a post hoc power calculation, because effect estimates and 95% CIs provide a more informative indication of estimate precision. All analyses were conducted in R, version 4.5.1 (R Foundation for Statistical Computing, Vienna, Austria).

## RESULTS

### Baseline Characteristics

During the study period, 17,327 patients presented to the ED. Of 14,440 enrolled patients, 10,531 were excluded, mainly due to discharge from the ED (9,439 patients). The final cohort included 3,909 admitted patients (Figure 1).

In these, median age was 72 years (IQR 55–83), and 52.8% were male. In-hospital mortality occurred in 168 patients (4.3%), 30-day mortality in 258 (6.6%), and 698 (17.9%) were admitted to the ICU; 76 (1.9%) were lost to follow-up and considered alive. Most patients were triaged as ESI level 2 (1,826/3,909; 46.7%) or level 3 (1,722/3,909; 44.1%). In-hospital mortality was highest in ESI level 1 (64/286; 22.4%), followed by level 2 (59/1,826; 3.2%) and level 3 (44/1,722; 2.6%), with similar patterns for 30-day mortality and ICU admission. In ESI level 4 and 5 patients (n = 75), adverse outcomes were rare, with one in-hospital death (1.3%) and four ICU admissions (5.3%). Most patients had a lactate < 2.0 mmol/L (2,641/3,909; 67.6%), followed by 2.0–3.9 mmol/L (1,041/3,909; 26.6%) and ≥ 4.0 mmol/L (227/3,909; 5.8%). In-hospital mortality increased with higher lactate levels: 2.3% (< 2.0 mmol/L), 4.8% (2.0–3.9 mmol/L), and 25.1% (≥ 4.0 mmol/L), again with similar patterns for 30-day mortality and ICU admission. Detailed distributions are presented in Table 1.

### Primary and Secondary Outcomes

Although crude in-hospital mortality was similar between patients triaged as ESI level 2 and 3 (3.2% and 2.6%, respectively), differences became more pronounced after stratification by lactate level. In ESI level 2 patients, in-hospital mortality increased from 2.0% among those with lactate < 2.0 mmol/L to 17.8% among those with lactate ≥ 4.0 mmol/L, corresponding to an age- and sex-adjusted absolute risk difference of 16.0% (95% CI, 8.8–23.3). In ESI level 3 patients, in-hospital mortality increased from 1.6% at lactate < 2.0 mmol/L to 15.1% at lactate ≥ 4.0 mmol/L, corresponding to a risk difference of 12.5% (95% CI, 3.7–21.3) (Table 2, Figure 2).

Among patients without lactate measurements, crude in-hospital mortality was also similar between ESI levels 2 and 3 (1.2% and 1.4%, respectively) (Table 3).

Across ESI levels 1–3 patients, lactate ≥ 4.0 mmol/L was associated with increased in-hospital and 30-day mortality, while associations with ICU admission varied by ESI level. In ESI level 1 and 2 patients, lactate values between 2.0–3.9 mmol/L were not associated with in-hospital mortality, 30-day mortality, or ICU admission. In contrast, in ESI level 3 patients, lactate values between 2.0–3.9 mmol/L were associated with in-hospital and 30-day mortality, but not with ICU admission (Tables 2 and 4).

Among the 1,722 patients triaged as ESI level 3, a lactate level ≥ 2 mmol/L captured 24 of 44 in-hospital deaths (54.5%) and 49 of 153 ICU admissions (32.0%), whereas a lactate level ≥ 4 mmol/L captured 8 of 44 in-hospital deaths (18.2%) and 8 of 153 ICU admissions (5.2%). The number of patients who would need to be flagged to capture one event was 19.5 vs 6.6 for in-hospital mortality and 9.6 vs 6.6 for ICU admission for the ≥ 2 mmol/L and ≥ 4 mmol/L thresholds (Supplementary Table S5). After adjustment for age and sex, models including both ESI and lactate showed higher discrimination for in-hospital mortality compared with ESI alone (AUC, 0.844 vs 0.799; ΔAUC, 0.045), with smaller differences for 30-day mortality (ΔAUC, 0.025) and ICU admission (ΔAUC, 0.006) (Table 5, Figure 3).

For in-hospital mortality, stratified analyses showed consistent benefits across age groups, with AUC increasing from 0.834 to 0.900 in patients < 65 years and from 0.746 to 0.796 in those ≥ 65 years (Supplementary Table S6).

## DISCUSSION

This study showed that among admitted ED patients who had lactate measured after triage at the treating physician’s discretion, higher lactate levels were associated with increased in-hospital mortality and 30-day mortality across ESI levels, whereas associations with ICU admission were weaker and varied by ESI level. Differences in outcomes across lactate categories were particularly evident among patients triaged as ESI levels 2 and 3, in whom overall mortality rates were similar.

The ability of the ESI to discriminate adverse outcomes is limited by its design as an acuity-based tool that relies in part on the subjective clinical judgment of triaging clinicians. In the present study, most patients were triaged as ESI level 2 or 3, which is consistent with previous findings.^4^,^28^ As expected, the highest in-hospital mortality was observed among patients triaged as ESI level 1. Among admitted patients with lactate measurements obtained after triage at the treating physician’s discretion, crude mortality rates for patients in ESI levels 2 and 3 were similar, at 3.2% and 2.6%, respectively. This may reflect differences in case mix and the selective use of lactate measurement after triage, as lactate was preferentially obtained in older and more severely ill patients, whereas those without lactate measurements were younger and had lower mortality (Supplementary Table S7). Notably, crude mortality rates were also similar between ESI levels 2 and 3 among admitted patients without lactate measurements (Table 3), supporting the interpretation that this pattern is not solely attributable to selection related to lactate measurement, but may also reflect limited discrimination of the ESI within these categories.

When stratified by lactate level, differences in mortality became more apparent. In ESI level 3 patients, a lactate threshold of ≥ 2 mmol/L identified a large proportion of patients who died, whereas a threshold of ≥ 4 mmol/L identified fewer patients with higher mortality. These findings suggest that lactate may help to further characterize differences in risk within this intermediate-acuity group.

These results are consistent with previous studies showing that higher lactate levels are associated with adverse outcomes and may provide additional information when considered alongside established clinical scores. For example, studies evaluating the National Early Warning Score (NEWS) have reported that higher lactate levels were associated with increased in-hospital mortality and ICU admission in general ED populations.^29^ Similarly, in patients with suspected sepsis, lactate ≥ 2 mmol/L has been associated with higher sensitivity for mortality or prolonged ICU stay when considered alongside qSOFA.^30^ Likewise, higher lactate levels have been associated with mortality across established severity scoring systems such as Acute Physiology and Chronic Health Evaluation II, SOFA, and Mortality in Emergency Department Sepsis in septic ED patients.^31^

In the present study, discrimination for 30-day mortality and ICU admission was lower than for in-hospital mortality, likely reflecting the impact of postadmission factors and institutional decision-making beyond the patient’s initial physiological condition. In older patients, previous studies have reported that lactate, when considered alongside different triage scales or clinical scores such as the Modified Early Warning Score (MEWS), NEWS, and qSOFA, was not associated with differences in discrimination of poor outcomes.^32^ In our study, however, increases in discrimination for in-hospital mortality were observed in older patients when lactate was considered alongside ESI, which may reflect differences in study populations and the selective use of lactate measurements.

The optimal lactate threshold associated with adverse outcomes in unselected adult ED patients remains uncertain, with reported cutoffs ranging from 2.0 to 4.0 mmol/L.^12^,^26^,^33^ In this study, even moderately elevated lactate levels (2.0–3.9 mmol/L) were associated with adverse outcomes, consistent with prior research.^34^ However, the association of intermediate lactate elevations with adverse outcomes differed across triage categories. While lactate levels of 2.0–3.9 mmol/L were not associated with adverse outcomes in ESI levels 1 and 2, they were associated with both in-hospital and 30-day mortality in ESI level 3 patients. This suggests that moderately elevated lactate levels may help to further characterize differences in risk within this heterogeneous triage level.

If lactate were measured systematically at the time of triage, it might enhance the discrimination of the ESI for patients ultimately admitted. In this context, point-of-care lactate testing may be of interest for identifying more subtle high-risk presentations, particularly among patients triaged as ESI level 3 who may otherwise appear clinically stable but could warrant more timely evaluation or closer monitoring. However, this potential role remains hypothetical and requires prospective evaluation.

## LIMITATIONS

Several limitations should be acknowledged. First, our study was conducted at a single tertiary care center in Europe, which limits its external validity. Second, the exclusion of discharged patients may have introduced selection bias toward a higher risk population, thereby limiting generalizability. In our cohort, 30-day mortality was 0.3% among discharged patients compared to 5.9% among admitted patients, consistent with previous reports.^35^ Third, lactate levels were measured at the treating physician’s discretion, potentially introducing selection bias. Patients without lactate measurements (and the small number with missing ESI) had lower rates of in-hospital mortality (1.4% vs 4.3%), 30-day mortality (2.8% vs 6.6%), and ICU admission (9.6% vs 17.9%) compared to included patients (Supplementary Tables S7 and S8), suggesting that less severely ill patients were underrepresented in the final cohort. Fourth, only the first lactate level was analyzed, without considering subsequent changes or lactate clearance, which may be more strongly associated with adverse outcomes than a single value.^36^–^38^ However, the aim was to evaluate the association of lactate during initial evaluation.

Fifth, the influence of potential confounding factors on lactate levels, such as alcohol consumption, liver disease, seizure, and medication, was not investigated and may have affected lactate measurements.^39^ Such information may be unavailable or unreliable during triage. Sixth, although lactic acidosis was previously reported to be more strongly associated with in-hospital mortality than hyperlactatemia, we did not differentiate between them to maintain a simple and clinically applicable approach.^40^,^41^ Seventh, details regarding prehospital care were not provided. Nevertheless, patients referred by general practitioners usually receive no prehospital treatment, and those brought in by emergency medical services have short transport times of less than 10 minutes due to the hospital’s central urban location. Eighth, the presence of hypotension was not analyzed separately. However, blood pressure is not included in the ESI algorithm version 4 to determine triage levels. In addition, previous studies have shown that the association between lactate levels and adverse outcomes is independent of blood pressure and the presence of shock.^24^,^42^

Finally, outcomes such as ICU admission may be influenced by factors including advance directives, institutional protocols, and resource availability. In addition, elevated lactate levels may directly influence clinical decision-making and disposition, since high lactate values are often perceived as markers of critical illness and may prompt ICU admission independent of the patient’s underlying physiological status. This may introduce confounding by indication and should be considered when interpreting associations between lactate levels and ICU admission.

## CONCLUSION

Among admitted ED patients with lactate measurements obtained after triage at the discretion of the treating physician, higher lactate levels were associated with in-hospital mortality across ESI levels, particularly in patients triaged as ESI levels 2 and 3. Given the single-center design, these findings should be validated in prospective studies across diverse ED settings.

## Supplementary Information





















## Figures and Tables

**Figure 1:**
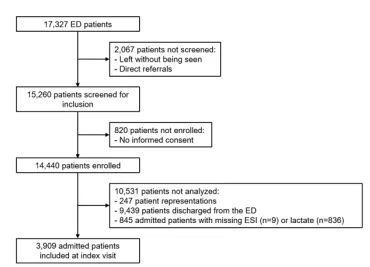
Flowchart of admitted adult emergency department patients with lactate measurements obtained after triage at physician discretion in a study that evaluated whether lactate levels were associated with in-hospital mortality across Emergency Severity Index levels. *ED*, emergency department; *ESI*, Emergency Severity Index.

**Figure 2:**
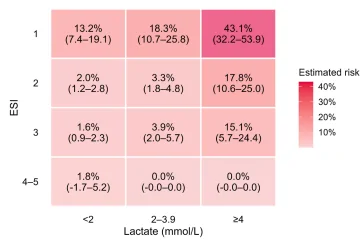
In-hospital mortality across Emergency Severity Index and lactate categories in admitted adult ED patients with lactate measurements obtained after triage at physician discretion, adjusted for age and sex. Values represent absolute risk estimates (95% confidence intervals) derived from logistic regression models. Confidence intervals extending below zero reflect limited precision due to small cell sizes in these strata. *ED*, emergency department; *ESI*, Emergency Severity Index.

**Figure 3:**
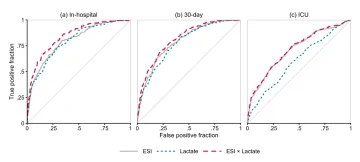
Receiver operating characteristic curves comparing age- and sex-adjusted models based on Emergency Severity Index (ESI) alone, lactate alone, and combined ESI–lactate model for (a) in-hospital mortality, (b) 30-day mortality, and (c) intensive care unit admission in admitted adult emergency department patients with lactate measurements obtained after triage at physician discretion. *ESI*, Emergency Severity Index; *ICU*, intensive care unit.

**Table 1:** Baseline characteristics and outcomes of admitted adult emergency department patients stratified by Emergency Severity Index and lactate category.

	N (%)	Age median (IQR)	Male %	In-hospital mortality n (%)	30-day mortality n (%) *	ICU admission n (%)
All patients	3,909	72 (55–83)	52.8	168/3,909 (4.3%)	258/3,909 (6.6%)	698/3,909 (17.9%)
ESI level 1	286/3,909 (7.3%)	71 (57–80)	59.8	64/286 (22.4%)	75/286 (26.2%)	171/286 (59.8%)
ESI level 2	1,826/3,909 (46.7%)	70 (54–81)	55.3	59/1,826 (3.2%)	100/1,826 (5.5%)	370/1,826 (20.3%)
ESI level 3	1,722/3,909 (44.1%)	75 (58–84)	49.7	44/1,722 (2.6%)	81/1,722 (4.7%)	153/1,722 (8.9%)
ESI level 4–5	75/3,909 (1.9%)	65 (41–82)	34.7	1/75 (1.3%)	2/75 (2.7%)	4/75 (5.3%)
Lac < 2.0 mmol/L	2,641/3,909 (67.6%)	72 (54–83)	50.4	61/2,641 (2.3%)	120/2,641 (4.5%)	401/2,641 (15.2%)
Lac 2.0–3.9 mmol/L	1,041/3,909 (26.6%)	73 (58–83)	57.1	50/1,041 (4.8%)	74/1,041 (7.1%)	213/1,041 (20.5%)
Lac ≥ 4 mmol/L	227/3,909 (5.8%)	71 (58–80)	59.9	57/227 (25.1%)	64/227 (28.2%)	84/227 (37.0%)

Data are presented for admitted adult emergency department patients with lactate measurements obtained after triage at physician discretion (N = 3,909).

* Patients lost to follow-up (n = 76) were considered alive.

*ESI*, Emergency Severity Index; *ICU*, intensive care unit; *IQR*, interquartile range; *Lac*, lactate.

**Table 2:** In-hospital mortality of admitted adult emergency department patients stratified by Emergency Severity Index and lactate category.

	N (%)	In-hospital mortality	
		n (%)	aOR (95% CI)
ESI level 1	286/3,909 (7.3%)	64/286 (22.4%)	
Lac < 2.0 mmol/L	121/286 (42.3%)	16/121 (13.2%)	Ref
Lac 2.0–3.9 mmol/L	93/286 (32.5%)	17/93 (18.3%)	1.30 (0.59–2.87)
Lac ≥ 4 mmol/L	72/286 (25.2%)	31/72 (43.1%)	5.27 (2.53–10.97)
ESI level 2	1,826/3,909 (46.7%)	59/1,826 (3.2%)	
Lac < 2.0 mmol/L	1,210/1,826 (66.3%)	24/1,210 (2.0%)	Ref
Lac 2.0–3.9 mmol/L	515/1,826 (28.2%)	17/515 (3.3%)	1.56 (0.82–2.95)
Lac ≥ 4 mmol/L	101/1,826 (5.5%)	18/101 (17.8%)	11.75 (6.01–22.97)
ESI level 3	1,722/3,909 (44.1%)	44/1,722 (2.6%)	
Lac < 2.0 mmol/L	1,254/1,722 (72.8%)	20/1,254 (1.6%)	Ref
Lac 2.0–3.9 mmol/L	415/1,722 (24.1%)	16/415 (3.9%)	2.22 (1.14–4.32)
Lac ≥ 4 mmol/L	53/1,722 (3.1%)	8/53 (15.1%)	10.57 (4.28–26.10)
ESI level 4–5	75/3,909 (1.9%)	1/75 (1.3%)	
Lac < 2.0 mmol/L	56/75 (74.7%)	1/56 (1.8%)	Ref
Lac 2.0–3.9 mmol/L	18/75 (24.0%)	0/18 (0.0%)	NA
Lac ≥ 4 mmol/L	1/75 (1.3%)	0/1 (0.0%)	NA

Data are presented for admitted adult emergency department patients with lactate measurements obtained after triage at physician discretion (N = 3,909). Adjusted odds ratios were estimated from age- and sex-adjusted logistic regression models. Within each ESI level, patients with lactate < 2.0 mmol/L served as the reference group. Adjusted odds ratios were not estimable for strata with zero events.

*aOR*, adjusted odds ratio; *ESI*, Emergency Severity Index; *Lac*, lactate; *Ref*, reference.

**Table 3:** Emergency Severity Index levels and in-hospital mortality stratified by lactate measurement status in admitted emergency department patients.

	All admitted patients (*N* = 4,745)		With lactate measurements (*n* = 3,909)		Without lactate measurements (*n* = 836)	

	*n* (%)	IHM, *n* (%)	*n* (%)	IHM, *n* (%)	*n* (%)	IHM, *n* (%)
ESI level 1	306/4,745 (6.4%)	67/306 (21.9%)	286/3,909 (7.3%)	64/286 (22.4%)	20/836 (2.4%)	3/20 (15%)
ESI level 2	1,994/4,745 (42.0%)	61/1,994 (3.1%)	1,826/3,909 (46.7%)	59/1,826 (3.2%)	168/836 (20.1%)	2/168 (1.2%)
ESI level 3	2,235/4,745 (47.1%)	51/2,235 (2.3%)	1,722/3,909 (44.1%)	44/1,722 (2.6%)	513/836 (61.4%)	7/513 (1.4%)
ESI level 4–5	210/4,745 (4.4%)	1/210 (0.5%)	75/3,909 (1.9%)	1/75 (1.3%)	135/836 (16.1%)	0/135 (0%)

Lactate measurements were obtained after triage at physician discretion. All patients refer to admitted index-visit patients with non-missing ESI; nine admitted patients with missing ESI were excluded from this ESI-stratified table.

*ESI*, Emergency Severity Index; *IHM*, in-hospital mortality.

**Table 4:** Thirty-day mortality and intensive care unit admission of admitted adult emergency department patients stratified by Emergency Severity Index and lactate category.

	*N* (%)	30-day mortality		ICU admission	
		*n* (%)	aOR (95% CI)	*n* (%)	aOR (95% CI)
ESI level 1	286/3,909 (7.3%)	75/286 (26.2%)		171/286 (59.8%)	
Lac < 2.0 mmol/L	121/286 (42.3%)	21/121 (17.4%)	Ref	65/121 (53.7%)	Ref
Lac 2.0–3.9 mmol/L	93/286 (32.5%)	21/93 (22.6%)	1.26 (0.60–2.65)	53/93 (57.0%)	1.06 (0.61–1.84)
Lac ≥ 4 mmol/L	72/286 (25.2%)	33/72 (45.8%)	4.47 (2.15–9.30)	53/72 (73.6%)	2.35 (1.24–4.46)
ESI level 2	1,826/3,909 (46.7%)	100/1,826 (5.5%)		370/1,826 (20.3%)	
Lac < 2.0 mmol/L	1,210/1,826 (66.3%)	55/1,210 (4.5%)	Ref	228/1,210 (18.8%)	Ref
Lac 2.0–3.9 mmol/L	515/1,826 (28.2%)	26/515 (5.0%)	1.02 (0.63–1.66)	119/515 (23.1%)	1.26 (0.98–1.62)
Lac ≥ 4 mmol/L	101/1,826 (5.5%)	19/101 (18.8%)	5.52 (3.02–10.12)	23/101 (22.8%)	1.23 (0.75–1.99)
ESI level 3	1,722/3,909 (44.1%)	81/1,722 (4.7%)		153/1,722 (8.9%)	
Lac < 2.0 mmol/L	1,254/1,722 (72.8%)	42/1,254 (3.3%)	Ref	104/1,254 (8.3%)	Ref
Lac 2.0–3.9 mmol/L	415/1,722 (24.1%)	27/415 (6.5%)	1.80 (1.09–2.97)	41/415 (9.9%)	1.19 (0.81–1.75)
Lac ≥ 4 mmol/L	53/1,722 (3.1%)	12/53 (22.6%)	8.78 (4.13–18.69)	8/53 (15.1%)	1.89 (0.87–4.11)
ESI level 4–5	75/3,909 (1.9%)	2/75 (2.7%)		4/75 (5.3%)	
Lac < 2.0 mmol/L	56/75 (74.7%)	2/56 (3.6%)	Ref	4/56 (7.1%)	Ref
Lac 2.0–3.9 mmol/L	18/75 (24.0%)	0/18 (0.0%)	NA	0/18 (0.0%)	NA
Lac ≥ 4 mmol/L	1/75 (1.3%)	0/1 (0.0%)	NA	0/1 (0.0%)	NA

Data are presented for admitted adult emergency department patients with lactate measurements obtained after triage at physician discretion (N = 3,909). Adjusted odds ratios were estimated from age- and sex-adjusted logistic regression models. Within each ESI level, patients with lactate < 2.0 mmol/L served as the reference group. Adjusted odds ratios were not estimable for strata with zero events.

*aOR*, adjusted odds ratio; *ESI*, Emergency Severity Index; *ICU*, intensive care unit; *Lac*, lactate; *Ref*, reference.

**Table 5:** Discriminatory performance of Emergency Severity Index (ESI) and ESI–lactate models in admitted adult emergency department patients with lactate measurements obtained after triage at physician discretion.

Outcome	AUC (95% CI): ESI	AUC (95% CI): Lac	AUC (95% CI): ESI×Lac	ΔAUC (95% CI)	LR *χ*^2^	LR P
In-hospital	0.799 (0.76–0.83)	0.794 (0.76–0.83)	0.844 (0.81–0.87)	0.045 (0.021–0.069)	88.43	< .001
30-day	0.791 (0.76–0.82)	0.776 (0.75–0.80)	0.816 (0.79–0.84)	0.025 (0.009–0.041)	74.85	< .001
ICU	0.709 (0.69–0.73)	0.603 (0.58–0.63)	0.715 (0.69–0.74)	0.006 (0.001–0.012)	16.94	.03

AUCs are derived from age- and sex-adjusted logistic regression models. ΔAUC indicates the change in AUC after adding lactate to the ESI. Likelihood-ratio tests compare nested ESI-only and combined ESI×lactate logistic regression models.

*AUC*, area under the curve; χ^2^, chi-square statistic; *ESI*, Emergency Severity Index; *ICU*, intensive care unit; *LR*, likelihood ratio; *Lac*, lactate.
