# Supplementary material for: Emergency Severity Index Stratified by Post-Triage Lactate: Association with In-Hospital Mortality in Admitted Patients
Source: West J Emerg Med. 2026 Aug 23;27(5):1364–73. doi: 10.5811/westjem.62115 (PMC13660567; doi:10.5811/westjem.62115)
Supplement: Supplementary file 1 [file wjem-27-1364-s001.docx]

**Figure S1.** Receiver operating characteristic curves comparing unadjusted models based on ESI alone, lactate alone, and combined ESI–lactate model for (a) in-hospital mortality, (b) 30-day mortality, and (c) ICU admission in admitted adult ED patients with lactate measurements obtained after triage at physician discretion.

Abbreviations: ED = emergency department; ESI = Emergency Severity Index; ICU = intensive care unit.
