# Supplementary material for: Emergency Severity Index Stratified by Post-Triage Lactate: Association with In-Hospital Mortality in Admitted Patients
Source: West J Emerg Med. 2026 Aug 23;27(5):1364–73. doi: 10.5811/westjem.62115 (PMC13660567; doi:10.5811/westjem.62115)
Supplement: Supplementary file 2 [file wjem-27-1364-s002.docx]

**Table S1.** Sensitivity analyses for in-hospital and 30-day mortality in admitted adult ED patients with lactate measurements obtained after triage at physician discretion.

| **Outcome** | **Sensitivity** | **AUC (95% CI)** | **ΔAUC (95% CI)** | **Notes** |
| --- | --- | --- | --- | --- |
| In-hospital mortality | Splines: ns(lactate) + ns(age) | 0.848 (0.82–0.88) | 0.004 (-0.006–0.013) | Knots lactate 0.80/1.30/1.90/4.10; age 29/63/79/91 |
| In-hospital mortality | Temporal drift | 0.852 (0.82–0.88) | 0.008 (-0.002–0.017) | 4 EMERGE waves; models refit on N=3,909 |
| 30-day mortality | LTFU excluded | 0.815 (0.79–0.84) | -0.001 (-0.037–0.035) | Excluded LTFU n=76; ref N=3,909, excl N=3,833 |
| 30-day mortality | LTFU set to deaths | 0.722 (0.69–0.75) | -0.094 (-0.134–-0.054) | Reclassified LTFU n=76 as deaths; fit on N=3,909 |

Sensitivity analyses assessing the robustness of AUC estimates for in-hospital and 30-day mortality. Analyses included modeling non-linearity using restricted cubic splines for lactate and age, adjustment for temporal drift across EMERGE waves, and alternative handling of 30-day LTFU. ΔAUC reflects the change in discrimination after adding lactate to the ESI. AUCs are derived from age- and sex-adjusted logistic regression models; values are reported relative to the reference model of each analysis (in-hospital mortality: primary age- and sex-adjusted model; 30-day mortality: model with LTFU coded as survivors).

Abbreviations: AUC = area under the curve; ΔAUC = change in AUC; CI = confidence interval; ED = emergency department; LTFU = lost to follow-up; ns = natural spline.
