# Supplementary material for: Emergency Severity Index Stratified by Post-Triage Lactate: Association with In-Hospital Mortality in Admitted Patients
Source: West J Emerg Med. 2026 Aug 23;27(5):1364–73. doi: 10.5811/westjem.62115 (PMC13660567; doi:10.5811/westjem.62115)
Supplement: Supplementary file 3 [file wjem-27-1364-s003.docx]

**Table S2.** Unadjusted in-hospital mortality of adult ED patients stratified by ESI and lactate category.

|  | **N (%)** | **In-hospital mortality** | |
| --- | --- | --- | --- |
|  |  | **n (%)** | **OR (95% CI)** |
| **ESI level 1** | 286/3,909 (7.3%) | 64/286 (22.4%) |  |
| Lac <2.0 mmol/L | 121/286 (42.3%) | 16/121 (13.2%) | Ref |
| Lac 2.0–3.9 mmol/L | 93/286 (32.5%) | 17/93 (18.3%) | 1.47 (0.70–3.09) |
| Lac ≥4 mmol/L | 72/286 (25.2%) | 31/72 (43.1%) | 4.96 (2.45–10.03) |
|  |  |  |  |
| **ESI level 2** | 1,826/3,909 (46.7%) | 59/1,826 (3.2%) |  |
| Lac <2.0 mmol/L | 1,210/1,826 (66.3%) | 24/1,210 (2.0%) | Ref |
| Lac 2.0–3.9 mmol/L | 515/1,826 (28.2%) | 17/515 (3.3%) | 1.69 (0.90–3.17) |
| Lac ≥4 mmol/L | 101/1,826 (5.5%) | 18/101 (17.8%) | 10.72 (5.59–20.56) |
|  |  |  |  |
| **ESI level 3** | 1,722/3,909 (44.1%) | 44/1,722 (2.6%) |  |
| Lac <2.0 mmol/L | 1,254/1,722 (72.8%) | 20/1,254 (1.6%) | Ref |
| Lac 2.0–3.9 mmol/L | 415/1,722 (24.1%) | 16/415 (3.9%) | 2.47 (1.27–4.83) |
| Lac ≥4 mmol/L | 53/1,722 (3.1%) | 8/53 (15.1%) | 10.97 (4.58–26.27) |
|  |  |  |  |
| **ESI level 4**–**5** | 75/3,909 (1.9%) | 1/75 (1.3%) |  |
| Lac <2.0 mmol/L | 56/75 (74.7%) | 1/56 (1.8%) | Ref |
| Lac 2.0–3.9 mmol/L | 18/75 (24.0%) | 0/18 (0.0%) | NA |
| Lac ≥4 mmol/L | 1/75 (1.3%) | 0/1 (0.0%) | NA |

Data are presented for admitted adult ED patients with lactate measurements obtained after triage at physician discretion (N = 3,909). ORs are unadjusted. Within each ESI level, patients with lactate <2.0 mmol/L served as the reference group. ORs were not estimable for strata with zero events.

Abbreviations: CI = confidence interval; ED = emergency department; ESI = Emergency Severity Index; Lac = lactate; OR = odds ratio; Ref = reference.
