# Supplementary material for: Emergency Severity Index Stratified by Post-Triage Lactate: Association with In-Hospital Mortality in Admitted Patients
Source: West J Emerg Med. 2026 Aug 23;27(5):1364–73. doi: 10.5811/westjem.62115 (PMC13660567; doi:10.5811/westjem.62115)
Supplement: Supplementary file 4 [file wjem-27-1364-s004.docx]

**Table S3.** Unadjusted 30-day mortality and ICU admission of admitted adult ED patients stratified by ESI and lactate category.

|  | **N (%)** | **30-day mortality** | | **ICU admission** | |
| --- | --- | --- | --- | --- | --- |
|  |  | **n (%)** | **OR (95% CI)** | **n (%)** | **OR (95% CI)** |
| **ESI level 1** | 286/3,909 (7.3%) | 75/286 (26.2%) |  | 171/286 (59.8%) |  |
| Lac <2.0 mmol/L | 121/286 (42.3%) | 21/121 (17.4%) | Ref | 65/121 (53.7%) | Ref |
| Lac 2.0–3.9 mmol/L | 93/286 (32.5%) | 21/93 (22.6%) | 1.39 (0.71–2.73) | 53/93 (57.0%) | 1.14 (0.66–1.97) |
| Lac ≥4 mmol/L | 72/286 (25.2%) | 33/72 (45.8%) | 4.03 (2.08–7.81) | 53/72 (73.6%) | 2.40 (1.27–4.54) |
|  |  |  |  |  |  |
| **ESI level 2** | 1,826/3,909 (46.7%) | 100/1,826 (5.5%) |  | 370/1,826 (20.3%) |  |
| Lac <2.0 mmol/L | 1,210/1,826 (66.3%) | 55/1,210 (4.5%) | Ref | 228/1,210 (18.8%) | Ref |
| Lac 2.0–3.9 mmol/L | 515/1,826 (28.2%) | 26/515 (5.0%) | 1.12 (0.69–1.80) | 119/515 (23.1%) | 1.29 (1.01–1.66) |
| Lac ≥4 mmol/L | 101/1,826 (5.5%) | 19/101 (18.8%) | 4.87 (2.76–8.59) | 23/101 (22.8%) | 1.27 (0.78–2.07) |
|  |  |  |  |  |  |
| **ESI level 3** | 1,722/3,909 (44.1%) | 81/1,722 (4.7%) |  | 153/1,722 (8.9%) |  |
| Lac <2.0 mmol/L | 1,254/1,722 (72.8%) | 42/1,254 (3.3%) | Ref | 104/1,254 (8.3%) | Ref |
| Lac 2.0–3.9 mmol/L | 415/1,722 (24.1%) | 27/415 (6.5%) | 2.01 (1.22–3.30) | 41/415 (9.9%) | 1.21 (0.83–1.77) |
| Lac ≥4 mmol/L | 53/1,722 (3.1%) | 12/53 (22.6%) | 8.45 (4.14–17.25) | 8/53 (15.1%) | 1.97 (0.90–4.29) |
|  |  |  |  |  |  |
| **ESI level 4**–**5** | 75/3,909 (1.9%) | 2/75 (2.7%) |  | 4/75 (5.3%) |  |
| Lac <2.0 mmol/L | 56/75 (74.7%) | 2/56 (3.6%) | Ref | 4/56 (7.1%) | Ref |
| Lac 2.0–3.9 mmol/L | 18/75 (24.0%) | 0/18 (0.0%) | NA | 0/18 (0.0%) | NA |
| Lac ≥4 mmol/L | 1/75 (1.3%) | 0/1 (0.0%) | NA | 0/1 (0.0%) | NA |

Data are presented for admitted adult ED patients with lactate measurements obtained after triage at physician discretion (N = 3,909). ORs are unadjusted. Within each ESI level, patients with lactate <2.0 mmol/L served as the reference group. ORs were not estimable for strata with zero events.

Abbreviations: CI = confidence interval; ED = emergency department; ESI = Emergency Severity Index; ICU = intensive care unit; Lac = lactate; OR = odds ratio; Ref = reference.
