# Supplementary material for: Emergency Severity Index Stratified by Post-Triage Lactate: Association with In-Hospital Mortality in Admitted Patients
Source: West J Emerg Med. 2026 Aug 23;27(5):1364–73. doi: 10.5811/westjem.62115 (PMC13660567; doi:10.5811/westjem.62115)
Supplement: Supplementary file 5 [file wjem-27-1364-s005.docx]

**Table S4.** Unadjusted discriminatory performance of ESI and ESI–lactate models in admitted adult ED patients with lactate measurements obtained after triage at physician discretion.

| **Outcome** | **AUC (95% CI): ESI** | **AUC (95% CI): Lac** | **AUC (95% CI): ESI×Lac** | **ΔAUC (95% CI)** | **LR χ²** | **LR p** |
| --- | --- | --- | --- | --- | --- | --- |
| In-hospital | 0.681 (0.64–0.73) | 0.701 (0.66–0.74) | 0.768 (0.73–0.81) | 0.087 (0.048–0.126) | 88.41 | < .001 |
| 30-day | 0.633 (0.60–0.67) | 0.639 (0.60–0.67) | 0.700 (0.66–0.74) | 0.067 (0.033–0.101) | 73.24 | < .001 |
| ICU | 0.690 (0.67–0.71) | 0.570 (0.55–0.59) | 0.701 (0.68–0.72) | 0.011 (0.003–0.019) | 18.17 | .02 |

AUCs are derived from unadjusted models. ΔAUC indicates the change in AUC after adding lactate to the ESI. LR tests compare ESI-only models with combined ESI×lactate models.

Abbreviations: AUC = area under the curve; χ² = chi-square statistic; CI = confidence interval; ED = emergency department; ESI = Emergency Severity Index; ICU = intensive care unit; LR = likelihood ratio; Lac = lactate.
