# Supplementary material for: Emergency Severity Index Stratified by Post-Triage Lactate: Association with In-Hospital Mortality in Admitted Patients
Source: West J Emerg Med. 2026 Aug 23;27(5):1364–73. doi: 10.5811/westjem.62115 (PMC13660567; doi:10.5811/westjem.62115)
Supplement: Supplementary file 6 [file wjem-27-1364-s006.docx]

**Table S5.** Lactate-based flagging rules for in-hospital mortality and ICU admission among admitted adult ED patients triaged as ESI level 3 with lactate measurements obtained after triage at physician discretion.

| **Rule** | **Flagged**  **(n, %)** | **IHM in flagged (n, %)** | **IHM captured**  **(n, % of all IHM)** | **N flagged per IHM captured** | **ICU in flagged (n, %)** | **ICU captured**  **(n, % of all ICU)** | **N flagged per**  **ICU captured** |
| --- | --- | --- | --- | --- | --- | --- | --- |
| Lac ≥2 mmol/L | 468/1,722 (27.2%) | 24/468  (5.1%) | 24/44  (54.5%) | 19.5 | 49/468  (10.5%) | 49/153  (32.0%) | 9.6 |
| Lac ≥4 mmol/L | 53/1,722 (3.1%) | 8/53  (15.1%) | 8/44  (18.2%) | 6.6 | 8/53  (15.1%) | 8/153  (5.2%) | 6.6 |

Lactate-based flagging rules (≥2 mmol/L and ≥4 mmol/L) among ESI level 3 patients (N = 1,722) are shown with the number and proportion of patients flagged, the number and proportion of IHM and ICU admissions among flagged patients, and the proportion of all IHM and ICU admissions captured by each rule. “N flagged per IHM captured” and “N flagged per ICU captured” indicate the number of patients that would need to be flagged to identify one IHM or one ICU admission, respectively.

Abbreviations: ED = emergency department; ESI = Emergency Severity Index; ICU = intensive care unit; IHM = in-hospital mortality; Lac = lactate.
