# Supplementary material for: Emergency Severity Index Stratified by Post-Triage Lactate: Association with In-Hospital Mortality in Admitted Patients
Source: West J Emerg Med. 2026 Aug 23;27(5):1364–73. doi: 10.5811/westjem.62115 (PMC13660567; doi:10.5811/westjem.62115)
Supplement: Supplementary file 7 [file wjem-27-1364-s007.docx]

**Table S6.** Discriminatory performance of ESI and ESI–lactate models for in-hospital mortality in admitted adult ED patients stratified by age group.

| **Age group** | **N** | **AUC: ESI** | **AUC: ESI×Lac** | **ΔAUC (95% CI)** | **LR χ² (df)** | **LR p** |
| --- | --- | --- | --- | --- | --- | --- |
| <65 | 1,450 | 0.834 (0.74–0.93) | 0.900 (0.83–0.97) | 0.066 (-0.002–0.133) | 33.08 (8) | < .001 |
| ≥65 | 2,459 | 0.746 (0.70–0.79) | 0.796 (0.76–0.84) | 0.050 (0.018–0.082) | 62.89 (7) | < .001 |

Lactate measurements were obtained after triage at physician discretion. AUCs are derived from sex-adjusted logistic regression models. ΔAUC indicates the change in AUC after adding lactate to the ESI. LR tests compare ESI-only models with combined ESI×lactate models.

Abbreviations: AUC = area under the curve; χ² = chi-square statistic; CI = confidence interval; df = degrees of freedom; ED = emergency department; ESI = Emergency Severity Index; LR = likelihood ratio; Lac = lactate.
