# Supplementary material for: Emergency Severity Index Stratified by Post-Triage Lactate: Association with In-Hospital Mortality in Admitted Patients
Source: West J Emerg Med. 2026 Aug 23;27(5):1364–73. doi: 10.5811/westjem.62115 (PMC13660567; doi:10.5811/westjem.62115)
Supplement: Supplementary file 8 [file wjem-27-1364-s008.docx]

**Table S7.** Baseline characteristics and outcomes of adult ED patients who were discharged or admitted.

|  | **N (%)** | **Age**  **median (IQR)** | **Male**  **%** | **In-hospital mortality**  **n (%)** | **30-day mortality**  **n (%) *** | **ICU admission**  **n (%)** |
| --- | --- | --- | --- | --- | --- | --- |
| **Discharged patients** | 9,439 | 43 (30–59) | 52.4 | NA | 32/9,439 (0.3%)^3^ | NA |
| With complete data | 3,227/9,439 (34.2%) | 51 (35–67) | 49.7 | NA | 18/3,227 (0.6%) | NA |
| With missing data^1^ | 6,212/9,439 (65.8%) | 40 (28–55) | 53.7 | NA | 14/6,212 (0.2%) | NA |
|  |  |  |  |  |  |  |
| **Admitted patients** | 4,754 | 71 (53–82) | 52.0 | 180/4,754 (3.8%) | 282/4,754 (5.9%)^4^ | 779/4,754 (16.4%) |
| With complete data | 3,909/4,754 (82.2%) | 72 (55–83) | 52.8 | 168/3,909 (4.3%) | 258/3,909 (6.6%) | 698/3,909 (17.9%) |
| With missing data^2^ | 845/4,754 (17.8%) | 63 (44–79) | 48.4 | 12/845 (1.4%) | 24/845 (2.8%) | 81/845 (9.6%) |

Repeat visits were excluded (n = 247). * Patients lost to follow-up were considered alive. ICU admission and in-hospital mortality are not applicable to discharged patients.

Footnotes:

^1^ missing ESI n = 8, missing lactate n= 6204.

^2^ missing ESI n = 9, missing lactate n = 836.

^3^ lost to follow-up n = 455.

^4^ lost to follow-up n = 97.

Abbreviations: ED = emergency department; ESI = Emergency Severity Index; ICU = intensive care unit; IQR = interquartile range.
