# Supplementary material for: Emergency Severity Index Stratified by Post-Triage Lactate: Association with In-Hospital Mortality in Admitted Patients
Source: West J Emerg Med. 2026 Aug 23;27(5):1364–73. doi: 10.5811/westjem.62115 (PMC13660567; doi:10.5811/westjem.62115)
Supplement: Supplementary file 9 [file wjem-27-1364-s009.docx]

**Table S8.** Baseline characteristics and outcomes of admitted adult ED patients stratified by inclusion status and EMERGE cohort.

|  | **N (%)** | **Age**  **median (IQR)** | **Male**  **%** | **In-hospital mortality**  **n (%)** | **30-day mortality**  **n (%)** | **ICU admission**  **n (%)** |
| --- | --- | --- | --- | --- | --- | --- |
| **Admitted patients** | 4,754 | 71 (53–82) | 52.0 | 180/4,754 (3.8%) | 282/4,754 (5.9%) | 779/4,754 (16.4%) |
| Included patients | 3,909/4,754 (82.2%) | 72 (55–83) | 52.8 | 168/3,909 (4.3%) | 258/3,909 (6.6%) | 698/3,909 (17.9%) |
| Excluded patients^1^ | 845/4,754 (17.8%) | 63 (44–79) | 48.4 | 12/845 (1.4%) | 24/845 (2.8%) | 81/845 (9.6%) |
|  |  |  |  |  |  |  |
| **EMERGE cohort 1**^a^ | 648/4,754 (13.6%) | 70 (52–82) | 50.9 | 21/648 (3.2%) | 34/648 (5.2%) | 126/648 (19.4%) |
| Included patients | 441/648 (68.1%) | 72 (57–83) | 51.0 | 17/441 (3.9%) | 27/441 (6.1%) | 89/441 (20.2%) |
| Excluded patients^2^ | 207/648 (31.9%) | 63 (45–79) | 50.7 | 4/207 (1.9%) | 7/207 (3.4%) | 37/207 (17.9%) |
|  |  |  |  |  |  |  |
| **EMERGE cohort 2**^b^ | 869/4,754 (18.3%) | 72 (54–83) | 51.0 | 50/869 (5.6%) | 65/869 (7.5%) | 170/869 (19.6%) |
| Included patients | 681/869 (78.4%) | 73 (57–83) | 53.5 | 46/681 (6.8%) | 58/681 (8.5%) | 151/681 (22.2%) |
| Excluded patients^3^ | 188/869 (21.6%) | 67 (45–81) | 42.0 | 4/188 (2.1%) | 7/188 (3.7%) | 19/188 (10.1%) |
|  |  |  |  |  |  |  |
| **EMERGE cohort 3**^c^ | 822/4,754 (17.3%) | 73 (54–83) | 51.7 | 35/822 (4.3%) | 55/822 (6.7%) | 134/822 (16.3%) |
| Included patients | 700/822 (85.2%) | 73 (56–84) | 52.3 | 33/700 (4.7%) | 51/700 (7.3%) | 125/700 (17.9%) |
| Excluded patients^4^ | 122/822 (14.8%) | 67 (47–79) | 48.4 | 2/122 (1.6%) | 4/122 (3.3%) | 9/122 (7.4%) |
|  |  |  |  |  |  |  |
| **EMERGE cohort 4**^d^ | 2,415/4,754 (50.8%) | 70 (53–82) | 52.7 | 74/2,415 (3.1%) | 128/2,415 (5.3%) | 349/2,415 (14.5%) |
| Included patients | 2,087/2,415 (86.4%) | 72 (55–82) | 53.0 | 72/2,087 (3.4%) | 122/2,087 (5.8%) | 333/2,087 (16.0%) |
| Excluded patients^5^ | 328/2,415 (13.6%) | 58 (41–77) | 50.6 | 2/328 (0.6%) | 6/328 (1.8%) | 16/328 (4.9%) |

Included and excluded patients refer to availability of complete data. Percentages are calculated within each stratum.

Footnotes:

^1^ missing ESI n = 9, missing lactate n = 836.

^2^ missing ESI n = 5, missing lactate n = 202.

^3^ missing ESI n = 4, missing lactate n = 184.

^4^ missing ESI n = 0, missing lactate n = 122.

^5^ missing ESI n = 0, missing lactate n = 328.

^a^ time of data acquisition from 21 October to 11 November 2013.

^b^ time of data acquisition from 1 February to 23 February 2015.

^c^ time of data acquisition from 30 January to 19 February 2017.

^d^ time of data acquisition from 18 March to 20 May 2019.

Abbreviations: ED = emergency department; ESI = Emergency Severity Index; ICU = intensive care unit; IQR = interquartile range.
